# Progressive microbial alterations in the human gut and lung across early life: implications for translational medicine

**DOI:** 10.3389/fcimb.2026.1739520

**Published:** 2026-06-04

**Authors:** Yaping Zhao, Yuan Gao, Xiao Li, Elizabeth M. Georgian, Kent E. Pinkerton, Chuanzhen Zhang

**Affiliations:** 1 Department of Gastroenterology, The First Affiliated Hospital of Shandong First Medical University & Shandong Provincial Qianfoshan Hospital, Jinan, Shandong, China; 2 School of Clinical Medicine, Shandong Second Medical University, Weifang, Shandong, China; 3 Western Center for Agricultural Health and Safety, University of California, Davis, Davis, CA, United States; 4 Center for Health and the Environment, University of California, Davis, Davis, CA, United States

**Keywords:** first 1000 days of life, gut microbiota, gut-lung axis, lung microbiota, translational medicine

## Abstract

Early life represents a critical window for the establishment and maturation of the human microbiome. The sterile womb paradigm remains dominant in the field, but *in utero* microbial colonization is highly controversial due to challenges related to low-biomass contamination. After birth, the intestinal and pulmonary microbial communities undergo a programmed succession shaped by factors such as delivery mode, feeding practices, and environmental exposures, and they bidirectionally regulate host immune and metabolic homeostasis via the gut–lung axis. Dysbiosis during early life is closely associated with allergic diseases, metabolic disorders, and respiratory illnesses. This review summarizes the dynamic succession patterns of the intestinal and pulmonary microbiota during early life, analyzes the methodological origins of the ongoing controversy over *in utero* colonization, delineates the developmental characteristics of the pulmonary microbiome at distinct stages, and systematically discusses the translational potential, clinical risks, and ethical challenges of microbiome-targeted interventions. Collectively, this review aims to provide a theoretical reference for early-life health protection and disease prevention.

## 1 Introduction

The human microbiota, comprising microorganisms and their metabolites, is a key determinant of host homeostasis (Berg et al., 2020). Its composition and diversity are critical to physiology, immune regulation, metabolism, and pathogen resistance (Kosiewicz et al., 2011). As such, the microbiome serves as a key biomarker of human health (Hou et al., 2022). The intricate host-microbiome interactions are pivotal in biomedical research, playing a crucial role in advancing translational medicine and offering new insights into disease prevention and treatment (Gilbert et al., 2025). By bridging basic science with clinical practice, this field offers innovative strategies for disease prevention, diagnosis, and treatment (Bautista et al., 2026).

The human immune system and the microbiome engage in a dynamic interplay that maintains immune homeostasis (Belkaid and Harrison, 2017). Increasing evidence suggests that microbiota residing on various body surfaces, such as the gastrointestinal tract, lungs, skin, oral cavity, and vagina, play a crucial role in modulating the host immune system, influencing both local and systemic tissues (Wang et al., 2023a; Heidari et al., 2024). The broad immunomodulatory effects of the microbiome are particularly evident in the interactions between the gut and respiratory tracts (Budden et al., 2017).

The human gastrointestinal tract harbors over 10¹³ symbiotic microorganisms, forming a complex ecosystem dominated by bacteria (Sender et al., 2016). Considerable research has focused on the role of the gut microbiota in regulating key host pathways, including systemic and local immunity as well as metabolism (Krutzik et al., 2005; Papa et al., 2024). The gut microbiota and host co-develop throughout life, and as microbial composition is shaped by various intrinsic and extrinsic factors (Planer et al., 2016; Korpela and de Vos, 2018), its dynamics can reveal critical links between host homeostasis and disease risk. Therefore, understanding how microbial alterations influence host physiology is crucial for developing novel therapeutics for immune- and metabolic-related disorders, such as inflammatory bowel disease.

Lung microbiota research has long lagged behind that of the gut. Healthy lungs were once considered sterile, as reflected by their exclusion from the initial Human Microbiome Project. However, advances in sequencing have overturned this dogma, revealing a low-biomass yet diverse airway community in health (Li et al., 2024; Lipinksi et al., 2024). The advent of high-throughput molecular profiling around 2010 redefined airway microbiota composition and established respiratory microbiome science as a distinct discipline (Hilty et al., 2010). Unlike the gut, these communities possess limited self-sustaining capacity and distinct ecological dynamics (Whiteside et al., 2021), with major implications for chronic respiratory diseases and infections. These insights now underpin microbiome-targeted strategies to improve respiratory outcomes (Man et al., 2017; Li et al., 2022).

Recent studies on the gut-lung axis highlight the intestinal microbiota as a pivotal regulator of both gastrointestinal and pulmonary health, with implications for the treatment of various diseases (Iliano et al., 2020). Disruptions in the gut microbiota, or dysbiosis, are implicated in the progression of chronic respiratory diseases and infections, highlighting the potential of microbiota-targeted therapies to improve lung health (de Córdoba-Ansón et al., 2025).

**Figure 1:**
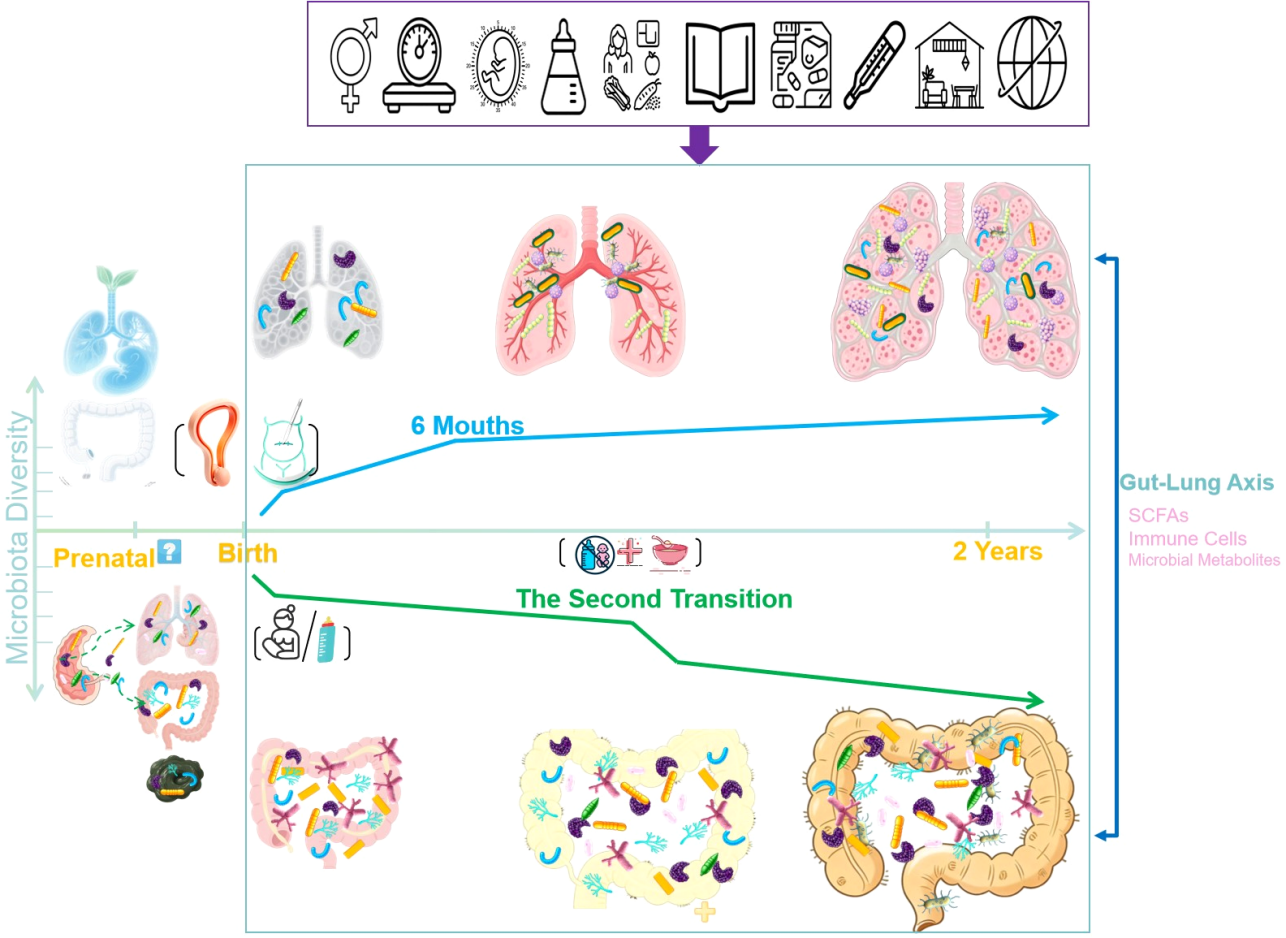
Key nodes of dynamic succession of gut and lung microbiota during the first 1000 days of life. This image illustrates the establishment, maturation, and coordinated succession of human gut and lung microbiota during the first 1000 days of life (conception to 2 years of age). It highlights the controversy of *in utero* colonization, major postnatal influencing factors, gut–lung axis crosstalk, and their links to host immune and metabolic homeostasis, and shows how delivery mode, feeding pattern, and complementary feeding shape microbial community structure and function.

This review synthesizes current knowledge on the assembly of the gut and lung microbiomes during the first 1,000 days of life, beginning from conception. We first evaluate the evidence for the sterile womb paradigm versus the *in utero* colonization hypothesis, and then describe the postnatal maturation and development of the infant microbiome. The synchronized development and crosstalk between these two microbial sites provide a foundation for microbiome — targeted strategies aimed at promoting lifelong health and preventing disease. Key developmental milestones are concisely summarized in **Figure 1**.

## 2 Methods

This review is based on published studies and aims to summarize the existing research on the gut and lung microbiota in fetuses, infants, and young children, highlight the relationship of the gut–lung axis, and outline relevant translational applications. A literature search was performed in PubMed, Google Scholar, and Web of Science. The search time range was from database inception to April 2026. The search strategy used Boolean operators (AND, OR) to combine the following keywords: “Gut microbiota”, “Lung microbiota”, “fetus”, “infant”,” young child”, “gut-lung axis”, and “Translational medicine”, covering both Medical Subject Headings (MeSH) and free-text terms. A total of 147 articles were included to support the viewpoints of this review.

## 3 Microbial establishment in the fetus

### 3.1 Sterile womb paradigm

Since Theodor Escherich first reported in 1885 that meconium contained no bacteria, healthy human fetuses were long considered sterile (Escherich, 1989). This led to the development of the sterile womb paradigm (Funkhouser and Bordenstein, 2013), which suggests that fetuses lack microbial colonization *in utero*, instead acquiring microorganisms during delivery or postnatally through vertical (i.e., from the mother) and horizontal (i.e., from other humans or the environment) transmission. This paradigm has been widely accepted for more than a century and serves as the fundamental framework for understanding the initial source of the neonatal microbiota. Despite recent challenges posed by sequencing technologies, multiple high-quality studies with strict contamination control still support the sterile womb paradigm as the current mainstream consensus in the field (Kennedy et al., 2023).

### 3.2 *In utero* colonization hypothesis

With the popularization of high-throughput molecular sequencing technologies, the *in utero* colonization hypothesis has been proposed, challenging the traditional sterile womb paradigm. This hypothesis states that the fetus is exposed to microbes prior to birth, and that low-abundance but biologically meaningful microbial communities exist in the placenta, amniotic fluid, meconium, and fetal lung, allowing initial colonization to occur prenatally (Stout et al., 2013; Aagaard et al., 2014).

#### 3.2.1 Placental microbiota

Bacterial DNA or bacteria-like structures have been detected in placenta, amniotic fluid, meconium, and other samples collected under strict aseptic conditions, and their presence has been confirmed using multiple techniques including cultivation. For instance, Stout et al. observed histological evidence of intracellular bacteria in the basal plate in nearly one-third of 195 placenta samples collected under strict sterile conditions (Stout et al., 2013). In a cohort of 320 participants, Aagaard et al. processed placental DNA under rigorous decontamination and aseptic environments and identified a unique placental microbial niche, with dominant phyla including *Firmicutes*, *Actinobacteria*, *Proteobacteria*, and *Fusobacteria*. 16S sequencing revealed that the placental microbiota profile was similar to that of the maternal oral microbiome, supporting the notion of microbial transmission from the maternal oral cavity to the fetus during pregnancy (Aagaard et al., 2014). Several studies have reported that placental microbial characteristics are independent of delivery mode, suggesting their prenatal origin (Jakobsson et al., 2014).

However, whether the placenta harbors an intrinsic microbiome remains a subject of debate, with different research groups reaching divergent conclusions based on their experimental data. Several studies support the traditional view of placental sterility. Using stringent negative controls combined with 16S rRNA gene sequencing and shotgun metagenomics, de Goffau et al. demonstrated that placental microbial signals mainly originate from reagent contamination or delivery-related exposure (de Goffau et al., 2019). Likewise, Panzer et al. showed that previously reported microbial signals were markedly reduced or disappeared altogether after effective contamination control (Panzer et al., 2023).

Although the existence of a placental microbiome is still a matter of debate, accumulating experimental evidence suggests that the maternal gut microbiota plays a regulatory role in placental and fetal development. Studies indicate that the maternal gut microbiota promotes placental development by modulating the metabolic milieu at the maternal–fetal interface, particularly via short-chain fatty acids (SCFAs) (Pronovost et al., 2023; Qin et al., 2024). Mechanistically, SCFAs stimulate tube formation in endothelial cell cultures and rescue placental vascularization defects in microbiota-depleted mice (Pronovost et al., 2023). In models of maternal malnutrition, gestational SCFA supplementation mitigates placental growth restriction and vascular dysfunction (Pronovost et al., 2023). Further supporting the gut–placenta axis, Zhang et al. demonstrated in a sheep model that gut microbiota dysbiosis mediates bisphenol A–induced intestinal and placental apoptosis, oxidative stress, and fetal growth restriction (Zhang et al., 2024). Collectively, these findings establish that gut microbiota-derived metabolites play a significant role in regulating maternal metabolism and immune responses, which can impact fetal development and pregnancy outcomes and are essential for maintaining placental health and function.

#### 3.2.2 Meconium microbiota

Meconium, the earliest stool of newborns, consists of swallowed amniotic fluid residues, bile acids, salts, and epithelial cells. Accumulating evidence indicates that meconium is not sterile and may undergo microbial colonization during gestation, consistent with observations that amniotic fluid itself could harbor a microbiota (Ardissone et al., 2014). Studies in humans have shown that the meconium microbiome exhibits reduced diversity, higher interindividual variability, enrichment of *Proteobacteria*, and decreased abundance of *Bacteroidetes* compared to adult feces (Dominguez-Bello et al., 2010). Experimental support from Jiménez et al. reinforces this notion: labeled *Enterococcus faecalis* was isolated from meconium of offspring born to dams orally inoculated with the same strain, suggesting maternal-fetal microbial translocation (Dominguez-Bello et al., 2010). Meconium microbiota composition also correlates with gestational age. Infants delivered before 33 weeks of gestation exhibit significantly reduced abundances of *Firmicutes*, *Actinobacteria*, *Proteobacteria* (excluding *Oxalicibacterium*), and *Bacteroidetes* compared to term infants (Ardissone et al., 2014). Certain genera, including *Enterobacter* and *Enterococcus*, have been further implicated in inflammatory processes associated with preterm birth (Stewart et al., 2012).

Collectively, these studies support the presence of a meconium microbiota of likely maternal origin, which may influence pregnancy outcomes. Nevertheless, this field remains controversial. Significant skepticism persists regarding the possibility that microbial signals originate from contamination rather than true colonization, a challenge particularly difficult to address in low-biomass samples such as meconium without stringent experimental controls (Salter et al., 2014; Rehbinder et al., 2018). However, issues including low microbial biomass, contamination interference, and the lack of viable bacterial validation leave the concept of meconium microbial colonization still controversial.

#### 3.2.3 Lung microbiota

Multiple lines of clinical evidence indicate that stable bacterial signals can be detected in the airway as early as the day of birth (Pammi et al., 2019). Lal et al. examined respiratory samples from extremely low birth weight (ELBW) preterm infants and full-term infants within 6 hours after birth, and found that both groups harbored microbial communities dominated by *Firmicutes* and *Proteobacteria*, accompanied by several other phyla including *Actinobacteria*, *Bacteroidetes*, *Tenericutes* (including *Ureaplasma*), and *Fusobacteria* (Lal et al., 2016). No significant difference was observed in α-diversity between the two groups, and their β-diversity was highly overlapping, suggesting that the airway microbiota is already present at birth with a similar basic composition (Lal et al., 2016; Pammi et al., 2019). Systematic reviews have also confirmed that bacterial signals can be detected in human neonates immediately or within hours after birth, and the bacterial load gradually increases with postnatal age (Pammi et al., 2019).

Regarding the speculation of possible prenatal exposure, current evidence remains mostly indirect and cannot confirm stable colonization. Gestational age and preterm status are key determinants of the early lung microbiota. Preterm infants, especially very preterm infants, often show reduced diversity, simplified community structure, and a predominance of *Staphylococcus* or *Ureaplasma* in the lower respiratory tract (Pattaroni et al., 2018). Pattaroni et al. further demonstrated that preterm infants with a gestational age of <30 weeks rarely develop a “mature mixed community” in the airway, indicating that gestational age is a central factor driving variation in the early postnatal lung microbiota (Pattaroni et al., 2018). The mainstream consensus in the field emphasizes that even if prenatal microbial exposure occurs, it is mostly low-abundance, transient, and non-stable; the substantial establishment and maturation of the neonatal airway microbiota occur primarily after birth.

### 3.3 The sterile womb paradigm and controversies over *in utero* colonization

Contamination control in low-biomass samples represents a major challenge in both supporting and opposing studies of *in utero* colonization. Microbial DNA content in fetal tissues is extremely low. For example, the median 16S rRNA gene copy number in amniotic fluid is approximately 67–207 copies/μL, with maximum values reaching thousands or higher, yet still markedly lower than that in fecal samples (approximately 1.5×10^7^ copies/μL) (Liu et al., 2025). Under such conditions, background bacterial DNA from reagent contamination, air exposure, sampling instruments, and laboratory environments can easily overwhelm genuine microbial signals.

The advantages and limitations of commonly used technical platforms for *in utero* and early respiratory microbiome research have been systematically reviewed in multiple methodological articles. These platforms differ considerably in applicability to low-biomass samples, contamination risk, and ability to distinguish viable bacteria (Knight et al., 2018; Eisenhofer et al., 2019), as summarized in **Table 1**.

**Table 1:** Advantages and limitations of common technical platforms for low-biomass microbiome analysis.

Technical platform	Advantages	Key limitations	Distinguishes viable bacteria from DNA fragments?	Eliminates reagent contamination?
16S-rRNA gene amplicon sequencing	Ultra-high sensitivity; detects extremely low-abundance DNA; low cost; suitable for large-scale screening	Cannot distinguish live/dead bacteria; amplification bias; no functional profiling; contamination signals easily amplified	No	Partially (depends on negative controls)
Shotgun metagenomics	Enables species- and strain-level identification; provides functional gene profiles; can infer cell viability via DNA damage patterns	High cost; sensitive to host DNA contamination (>99% host DNA in fetal tissues); still affected by low-biomass contamination	Partially (via DNA fragmentation analysis)	Partially
Bacterial culture	Only method to confirm viable colonization; allows antibiotic susceptibility and functional testing	Very low sensitivity (most bacteria unculturable); cannot detect non-viable or fastidious bacteria	Yes	Yes (aseptic collection + culture negative controls)
Fluorescence *in situ* hybridization (FISH)	Allows *in situ* visualization of bacterial morphology and spatial distribution; can be validated with confocal microscopy	Probe design relies on known sequences; interference from tissue autofluorescence; easy to miss low-abundance taxa; cannot assess viability	No (but intact cell morphology can be observed)	Yes (background control via tissue sections)
qPCR/digital PCR	Absolute quantification; high sensitivity; enables targeting of specific species	Cannot assess community diversity; cannot distinguish live/dead bacteria; results affected by primer desig	No	Partially

Notably, even when using identical molecular techniques (e.g., 16S rRNA gene sequencing), different studies can reach completely opposite conclusions. The main reasons include distinct bioinformatic filtering thresholds, variable decontamination pipelines, and inconsistent definitions of “colonization”—such as detection of DNA alone, verification of viable bacteria, or formation of a stable community. Some researchers regard the detection of microbial DNA as evidence of prenatal exposure, while others insist that viable bacteria must be confirmed, which directly leads to divergent interpretations (Tsou et al., 2020).

Therefore, as highlighted by Bihl et al., it is critical to use genomic features of independent biological samples—namely, unique profiles at the microbial strain level—as potential novel microbiome biomarkers to overcome technical challenges posed by low-biomass samples (Bihl et al., 2022).

## 4 Development of the microbiota in infants

The initial establishment of the infant microbiota, whether beginning *in utero* or postnatally, is followed by rapid exposure to a diverse microbial environment after the rupture of amniotic membranes. The primary maternal source of the infant microbiota is likely influenced by birth and early care. Early infant care is related to factors such as sex, birth weight, gestational age, mode of infant feeding, maternal diet, education, diseases, antibiotic exposure, familial environment, geographical location, and cultural traditions that participate in the formation of the neonatal microbiota (Butel et al., 2018; Xu et al., 2020).

The prolonged hospitalization of neonates has been strongly associated with gut microbiota dysbiosis, which is particularly marked by increased colonization by hospital-acquired pathogens (Goldmanln, 1981). A comparative cohort study revealed that extended postpartum hospital stays significantly increased the abundance of antimicrobial-resistant *Enterococcus* spp (Bashar et al., 2025). Intriguingly, geographical influences play a predominant role over conventional determinants, such as delivery modes and antibiotic exposure. Pan-European analyses demonstrated that regional variations accounted for 22% of microbiota variance (Fallani et al., 2010). Delivery modes, feeding patterns, and infant sex significantly shape the development of the infant gut microbiota (Ma et al., 2023).

### 4.1 Changes in the gut microbiota in infants

The establishment of a stable gut microbiota in infants involves two major transitional phases (Tanaka and Nakayama, 2017). The second transition takes place during the weaning period. With the introduction of solid foods, the gut microbiota gradually evolves into a complex, adult-like community.

#### 4.1.1 The first transition

The newborn begins to be colonized by symbiotic microorganisms during and immediately after birth. The early dominant colonizers of the infant gut are primarily facultative anaerobes, such as *Enterococcus faecalis*, *Escherichia coli*, *Staphylococcus* spp. Over time, the relative abundance of strict anaerobes increases. This shift is thought to reflect the transition of the gut environment from an aerobic to an anaerobic state (Bäckhed et al., 2015; Bittinger et al., 2020; Rao et al., 2021). The infant gut microbiota undergoes profound dynamic changes during the initial weeks and months of life. Recent investigations have revealed that the neonatal gut microbiota exhibits considerable diversity on the first day after delivery, accompanied by pronounced inter-individual heterogeneity in community structure and an absence of a stable configuration (Ma et al., 2023). These findings highlight the intricate regulatory mechanisms that govern early microbial colonization. Moreover, vertical transmission of maternal microorganisms, potential prenatal exposures in the intrauterine environment, and differential contact with environmental microbes postpartum collectively represent three pivotal determinants shaping early microbiome assembly (Ferretti et al., 2018). Supporting the importance of maternal transmission, Mitchell et al (Mitchell et al., 2020). recently demonstrated that bacterial strains colonizing the infant gut within the first week of life exhibited high concordance with those derived from the maternal rectal microbiota, independent of delivery mode or peripartum vaginal exposure. This observation supports the importance of maternal microbial vertical transmission in seeding the infant gut. Concurrently, a gradual decline in gut microbial diversity was observed over this period. The relative abundance of initially predominant commensals from maternal vaginal, oral, and skin niches consistently decreased, suggesting that these exogenous microbes may function as transient colonists participating in a dynamic successional process within the developing lower gastrointestinal tract. By the second week postpartum, the gut of infants delivered by cesarean section (CS) was notably depleted in *Bacteroides*—a bacterial group likely acquired during intrapartum transmission from mother to infant and subsequently diminished by antibiotic exposure (Rutayisire et al., 2016).

Delivery mode significantly influences the gut microbiota during the first three postnatal months, a critical window for immune development (Dogra et al., 2015). Compared to CS, vaginal delivery enriches the abundance and diversity of *Actinobacteria* and *Bacteroidetes* while reducing *Firmicutes* (Rutayisire et al., 2016). Vaginally delivered infants showed higher colonization levels of *Bifidobacterium* (Mitsou et al., 2008; Dogra et al., 2015) and *Bacteroides* (Jakobsson et al., 2014), whereas significantly more *Clostridium* (Hesla et al., 2014) and *Lactobacillus* (Kabeerdoss et al., 2013) colonized CS-delivered infants (Rutayisire et al., 2016). CS delivery has been linked to a range of adverse health outcomes. These include reduced levels of various cytokines and their receptors (Kabeerdoss et al., 2013), an elevated risk of neonatal opportunistic infections (Shao et al., 2019), immune disorders (Stokholm et al., 2018), obesity (Blustein et al., 2013), and neuroendocrine abnormalities, including cognitive and behavioral deficits associated with perturbations (Moya-Pérez et al., 2017) in the early-life microbiome. These associations are mainly due to the lack of specific microbial exposures during the CS procedure. It has been reported that restoring the maternal microbiota to infants delivered by cesarean section immediately after birth can normalize the developmental trajectory of their gut microbiota, suggesting a potential microbial-based intervention for mitigating these risks (Zhou et al., 2023).

#### 4.1.2 The second transition

Most infants are completely weaned from breast milk at around 1 year of age and transition to a solid food diet. This shift is accompanied by an increase in the alpha diversity of the gut microbiota and a transition toward an adult-like composition. Key changes include increased abundances of *Roseburia*, *Bifidobacterium*, *Clostridium*, *Bacteroides*, and *Anaerobacter*, along with an enrichment of genes involved in fiber degradation. Concurrently, total short-chain fatty acid (SCFA) levels rise, with a notable increase in butyrate. Furthermore, the composition of bifidobacteria shifts toward strains capable of metabolizing plant-derived polysaccharides (Bäckhed et al., 2015). During this weaning phase, microbial diversity increases significantly, and the differences previously associated with delivery mode gradually diminish (Dogra et al., 2015). Infants are exposed to a multitude of microorganisms from the environment and the solid foods they consume, which play an important role in altering the gut microbiota (Laursen et al., 2015; Tun et al., 2017; Amir et al., 2022; Carlino et al., 2024). Over time, the infant gut microbial configuration fluctuates, increasing in diversity and stability, especially during the first three years after birth (Yatsunenko et al., 2012). In humans, the maturation of the fecal microbiota is largely completed within the first few years of life, characterized by the typical establishment of *Firmicutes* and *Bacteroidetes* dominance by age 3 (Yatsunenko et al., 2012; Teo et al., 2018). A growing number of studies have focused on how the composition and development of early gut microbiota influence adult health, highlighting the transition from infancy to childhood as a critical window for targeted preventive strategies.

### 4.2 Changes in the lung microbiota with age

Early life, particularly the neonatal and infant periods, represents a critical window for the establishment and shaping of the lung microbiome. This process is closely associated with external environmental factors and the development and maturation of the respiratory and immune systems.

After birth, the infant immune system is immature and develops in response to environmental stimuli, while the lungs are exposed to the external environment for the first time. The source and composition of the lung microbiota are influenced by multiple factors, among which delivery mode is the primary determinant of the initial microbial source (Dominguez-Bello et al., 2010). Studies have shown that vaginally delivered neonates harbor early oropharyngeal microbiota similar to the maternal vaginal community, such as *Lactobacillus* and *Prevotella* (Dominguez-Bello et al., 2010). These microbes are inhaled during passage through the birth canal and form the foundation of the early respiratory microbiota. In contrast, cesarean section-born neonates bypass direct contact with the birth canal, and their initial microbiota is mainly derived from the maternal skin surface, such as *Staphylococcus* and *Propionibacterium* (Dominguez-Bello et al., 2010).

The lower respiratory tract was long considered sterile. With the development of high-throughput molecular techniques, it has become clear that similar to placental microbiome research, the lower respiratory tract in healthy lungs harbors an extremely low microbial biomass (Pérez-Cobas et al., 2023). Typically, bronchoalveolar lavage fluid (BALF) contains only 10^2^–10–^3^ mL^−1^, which is 2–3 orders of magnitude lower than in the upper respiratory tract (Dickson et al., 2017; Yagi et al., 2021). Against this low-biomass background, contamination signals from bronchoscope channels, reagents, and ambient air, as well as sampling bias from different anatomical sites and collection techniques, can easily mask genuine microbial signals and lead to false-positive or misleading conclusions (Drengenes et al., 2019). Therefore, methodological limitations must be fully considered when interpreting pediatric lung microbiome data to avoid overinterpretation.

Studies have shown that the lower respiratory tract microbiota overlaps with that of the upper respiratory tract, supporting the notion that upper respiratory tract and even oral contents enter the lower respiratory tract via direct downstream mucosal diffusion and microaspiration (Bassis et al., 2015; Kloepfer et al., 2018; Brewer et al., 2021). Thus, during the neonatal period, the microbiota is likely dominated by environmentally acquired and inhaled microbes.

After 6 months of age, the stability of the respiratory microbiome increases markedly, *Moraxella* gradually becomes the dominant genus, and microbial fluctuations decrease, while interindividual differences expand significantly (Biesbroek et al., 2014). As the immune system matures, the host’s ability to regulate the microbiota strengthens. Early microbiome colonization patterns can sustainably influence community stability and respiratory health outcomes (Pattaroni et al., 2018). At this stage, the associations among host immune maturation, microbial community homeostasis, and respiratory health become stronger. Crosstalk between the gut and respiratory microbiota (the gut–lung axis) may serve as a key regulatory mechanism and warrants further investigation in future studies.

### 4.3 Influence of the microbiota on homeostasis

**Figure 2:**
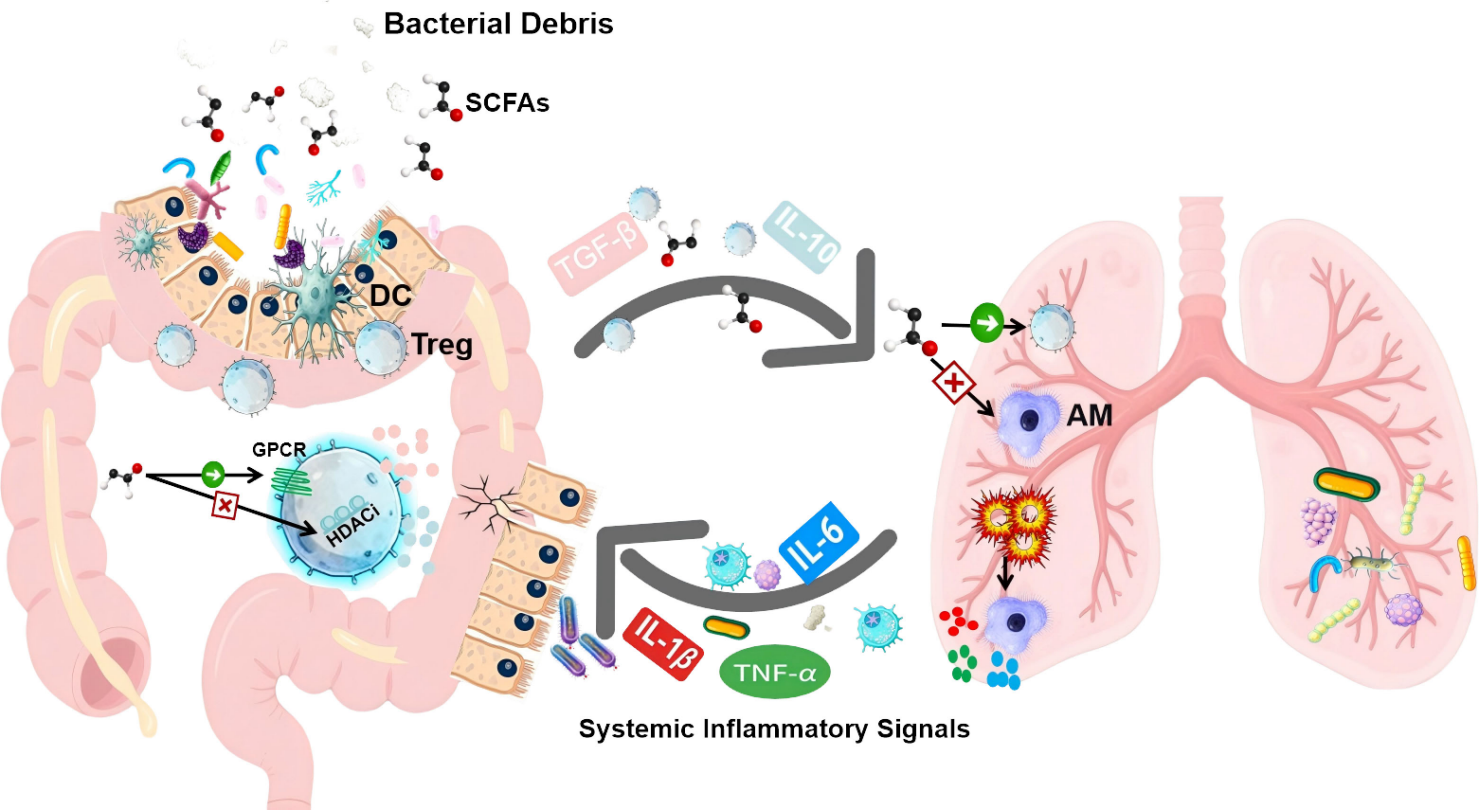
Schematic diagram of immune regulation mechanism of the gut-lung axis. This image illustrates the bidirectional communication of the gut–lung axis. Gut microbiota remotely regulates pulmonary immune responses via metabolites and immune mediators, while lung microbiota in turn modulates intestinal immunity and homeostasis.

Although the overall tissue architecture of the lung and gut is formed after birth, the epithelial barrier function remains immature. During this critical period, the commensal microbiota in the gut and lungs play indispensable roles in promoting organ maturation and driving the establishment of immune and metabolic homeostasis (Marsland et al., 2015). **Figure 2** illustrates the immunoregulation of the gut–lung axis, revealing how the gut microbiota modulates pulmonary immune responses and maintains immune homeostasis through metabolites and immune mediators.

#### 4.3.1 Effects of the gut microbiota

While the structure and function of a newborn’s GI tract are immature (Wagner et al., 2008), host–microbiota interactions promote their maturation. Early colonization by the gut microbiota significantly contributes to intestinal mucosal differentiation, barrier integrity, and immune tolerance (Shi et al., 2017). The gut microbiota exerts local and systemic regulatory effects through its bacterial components and metabolites, such as short-chain fatty acids (SCFAs) (Shi et al., 2017; McAleer and Kolls, 2018). SCFAs strengthen the intestinal barrier by enhancing the expression of epithelial tight junction proteins and stimulating mucin secretion. SCFAs, the main end products of dietary fiber fermented by the gut microbiota, play a central role in this process. SCFAs (including acetate, propionate, and butyrate) maintain host health through multiple mechanisms: (1) serving as an energy source for intestinal epithelial cells and supporting barrier function (Kim et al., 2016; Shao et al., 2022); (2) directly modulating immune cell function via G protein-coupled receptors (GPCRs) (Husted et al., 2017), promoting the recruitment and differentiation of immune cells such as regulatory T cells (Tregs) (Vinolo et al., 2011); and (3) epigenetically regulating gene expression and immune responses by inhibiting histone deacetylases (HDACs) (Thorburn et al., 2015). Dysregulation of these mechanisms, known as dysbiosis, is closely associated with an increased risk of later-life inflammatory diseases, such as inflammatory bowel disease (IBD) and asthma (Thorburn et al., 2015; Ihekweazu and Versalovic, 2018). Clinical studies are exploring SCFA-based therapeutic strategies.

Beyond these local intestinal effects, the gut microbiota profoundly influences host systemic metabolism and nutritional status. The early gut microbiota may also impact infant nutrition and growth by affecting growth hormones (Schwarzer et al., 2016). Infants fed primarily with mother’s own milk (MOM) exhibit increased gut microbiota diversity, along with higher abundances of *Bifidobacterium* and *Bacteroides*, which are significantly associated with a reduced risk of necrotizing enterocolitis (NEC) (Jin et al., 2019). In preterm infants with a birth weight <1,500 g (n=125), feeding with MOM for 6 weeks, compared with donor milk, was associated with *Bifidobacterium* and correlated with improved weight gain and overall growth (Ford et al., 2019). The gut microbiota may also be linked to metabolism, as its alteration due to infant antibiotic use may have long-term consequences, including obesity, insulin resistance, and liver diseases (Cox et al., 2014; Nobel et al., 2015; Mahana et al., 2016). Thus, the first 1,000 days represent a critical window for infant development, during which factors such as nutrition, environment, socioeconomics, and lifestyle influence the infant’s genome, health, and future disease risk (Shukla et al., 2017).

Of particular importance is the communication between the gut microbiota and the lungs via the “gut–lung axis.” Dysbiosis of the gut microbial community is related to lung disorders and infections (Trompette et al., 2014; Shukla et al., 2017). One study conducted on house dust mite (HDM)-challenged mice demonstrated significant associations between age-related gut microbial dysbiosis and allergic airway responses such as asthma (Vital et al., 2015). Additionally, recent studies indicate that differences in the gut microbiota during the first week of life are associated in infants and young children with clinically severe viral lower respiratory tract infections (vLRTIs) (Garcia-Mauriño et al., 2025). Similarly, an imbalance in the gut microbiota increases the risk of developing allergic responses and cystic fibrosis (Anand and Mande, 2018). The underlying mechanisms can be partially attributed to the systemic distribution of microbial metabolites: short-chain fatty acids (SCFAs) have been demonstrated to suppress pulmonary inflammatory responses. Meanwhile, other metabolites such as amino acid derivatives (ammonia, polyamines, hydrogen sulfide, phenol, and indole) exert multifaceted influences on the onset, progression, and therapeutic response of lung cancer (Teng et al., 2020; Vernocchi et al., 2020; Zhao et al., 2021). Fecal microbial transplants in humans have systemic effects, including beneficial impacts on respiratory diseases such as COPD (Jang et al., 2020). In summary, the gut microbiota serves as a pivotal nexus connecting the local intestinal milieu to systemic well-being, with special significance for lung health. It achieves this through regulating fundamental metabolic pathways such as energy metabolism and carbohydrate transport system signaling pathways (Kanehisa et al., 2008), while also governing the development and homeostasis of the immune system.

#### 4.3.2 Effects of the lung microbiota

The lung microbiota is believed to influence the maturation of alveolar morphology (Yun et al., 2014) and mucus production (Mathieu et al., 2018). Once the lung microbiota colonizes the local mucosal surface, pathogens are prevented from growing, provoking inflammation, and spreading (Bäumler and Sperandio, 2016). Unsurprisingly, the low number of immune cells and the immature phenotypes of these cells in newborns result in an attenuated and restricted immune response compared to that in adults, which largely determines the extent of airway hyperreactivity, mucus secretion, and susceptibility to reinfection (Chotirmall and Burke, 2015). While the lungs are remodeled and colonized by microbes during the postnatal period, immune cells accumulate slowly. As newborns mature, environmental factors, including premature birth, birth order, siblings, pets, dust, and cigarette smoke exposure, affect the development of the pulmonary immune system (Lloyd and Marsland, 2017). Tonic signals derived from early microbes are likely necessary for the maturation of lung-resident cells (e.g., alveolar macrophages [AMs] and CD11+ cells) via undefined mechanisms. The cytosine–phosphate–guanine (CpG) motif of bacterial DNA can activate the innate immune system via Toll–like receptors (Chuquimia et al., 2013; Kearney et al., 2015) or other pathogen recognition receptors present in AMs, as well as bronchial and alveolar epithelial cells (Chuquimia et al., 2012, Chuquimia et al., 2013).

Airway macrophages, which constitute the first line of defense against pathogens, colonize the airway 3–7 days after birth (Hashimoto et al., 2013; Tan and Krasnow, 2016) and self-renew under homeostatic conditions. The pulmonary epithelium secretes IgA, cytokines, and chemokines that collectively regulate its barrier function (e.g., ciliary action, mucin secretion) (Lloyd and Marsland, 2017). The number and function of adaptive immune cells in neonatal lungs are limited. In particular, T lymphocytes are present at a low frequency (Rennert et al., 1996) and are predominantly composed of naive T cells (Thome et al., 2016), which mount insufficient T-cell responses to bacterial or viral lung infections.

From a clinical standpoint, early variations in lung microbiota composition associated with delivery mode carry significant health implications. Compared with vaginally delivered infants, infants delivered by CS exhibit altered respiratory microbiota compositions and often show increased susceptibility to allergic asthma (Roduit et al., 2009). This finding is supported by a murine study comparing germ-free (GF) (n=6) and specific-pathogen-free (SPF) (n=11) neonatal mice by Remot et al (Remot et al., 2017). The authors showed that asthma and the lung microbiota reciprocally modulate each other and that bacterial stimuli within neonatal airways can protect against or worsen allergic asthma in youth (Remot et al., 2017). The murine lung microbiota plays a key role in the development of tolerance to aeroallergens early in life through the transient induction of PD-L1 expression in dendritic cells (DCs) and peripheral Treg cells (Gollwitzer et al., 2014). In the airways of healthy 1-month-old infants, the relative abundances of *Veillonella* and *Prevotella* were correlated with lower levels of proinflammatory airway immune mediators and higher levels of T-cell-recruiting chemoattractants. This immune profile is an independent predictor of asthma by age 6 (Kabeerdoss et al., 2013). Both of these specific lung bacteria are associated with more lymphocytes in BAL fluid, T helper (Th)17 cell-mediated lung inflammation, and a diminished TLR4 response by AMs (Man et al., 2017).

The lung microbiota not only modulates the development and function of local immune regulation but also interact with the GI tract, which is important for establishing local and systemic immune functions (Hooper et al., 2012). Changes in the lung microbiota also alter the composition of the gut microbiota (Sze et al., 2015) and intestinal immunity. Murine studies have shown that airway microbial dysbiosis caused by LPS leads to the translocation of lung bacteria into the bloodstream, increasing intestinal bacterial overload and disrupting gut microbial homeostasis (Sze et al., 2015). Some strains of pneumonia have also been shown to promote intestinal injury (Perrone et al., 2012). Pneumonia induced by *Pseudomonas aeruginosa* may induce intestinal injury by inhibiting gut epithelial proliferation and blocking the M phase of the cell cycle (Coopersmith et al., 2003; He et al., 2017). Hence, the microbiota influence the development of the immune system, which plays a central role in establishing and maintaining microbial communities (Rawls et al., 2006). Moreover, microorganisms enter the gut and lung through different mechanisms. Thus, the link between the gut and lung microbiota appears to be bidirectional and is stimulated by local and distal immune responses (Bingula et al., 2017; Budden et al., 2017).

## 5 Translational implications

The first 1,000 days of life represent a critical window for the development of early-life microbial communities and immune system maturation. Emerging evidence indicates that microbial modulation serves not only as a key strategy for preventing developmental disorders but also as an important target for promoting lifelong health through continuous interventions spanning the perinatal period (Orchanian and Hsiao, 2025; Strine and Konnikova, 2025). These findings provide new directions for clinical practice and public health strategies, while enhancing our understanding of the establishment processes of fetal and neonatal microbiota. However, most of these interventions remain at the research stage, and their translation from bench to bedside faces multiple challenges related to safety, standardization, and regulation.

### 5.1 Maternal interventions during pregnancy

Maternal interventions during pregnancy can reshape the microbial ecosystems of mothers and babies and have a profound impact on the long-term health of both mothers and their offspring (Tian et al., 2023; Alemu et al., 2024). Probiotic supplementation can improve maternal health, including immune function, as well as fetal immune function, thereby reducing the risk of pregnancy complications and promoting fetal development (Cuinat et al., 2022). Targeted modulation of the maternal microbiota during pregnancy has been demonstrated to have significant potential for improving fetal developmental programming (Davidson et al., 2021). Research has demonstrated that prenatal probiotic supplementation reduced the incidence of gestational diabetes by 62% through enhanced intestinal barrier integrity and downregulation of proinflammatory cytokines (Davidson et al., 2021). In addition, a recent prospective study demonstrated that the potentially protective fecal metabolites L-homoarginine (p=0.026) and vomifoliol (p=0.010) in mothers were positively correlated with maternal antibody levels and negatively correlated with the maximum body temperature of neonates infected with SARS-CoV-2, suggesting that maternal gut metabolites may play a role in regulating infant immune responses (Fu et al., 2024). Other studies have indicated that targeted modulation of the maternal gut microbiota during pregnancy, as well as supplementation with protective metabolites such as fatty acids and flavonoids, can regulate offspring immune development through the maternal-fetal axis and reduce the risk of atopic dermatitis in offspring (Du et al., 2025). Although these studies provide important clinical translational evidence for pregnancy interventions to improve maternal and child health, current studies generally have small sample sizes, high heterogeneity in intervention protocols (probiotic strains, dosages, timing of intervention), and most lack long-term offspring follow-up. Therefore, the evidence is insufficient to support universal clinical recommendations.

### 5.2 Perinatal and neonatal interventions

CS-associated microbial disturbances, including increased susceptibility to opportunistic infections, immune dysregulation, and neurodevelopmental impairments, have been mechanistically linked to adverse neonatal outcomes (Neu, 2017). To mitigate these risks, two evidence-based interventions have emerged: vaginal seeding and maternal fecal microbiota transplantation (FMT) (Dominguez-Bello et al., 2016; Korpela et al., 2020).

Vaginal seeding, a practice involving the topical application of maternal vaginal secretions to neonates born via cesarean section, aims to mimic the natural transfer of maternal microbiota that occurs during vaginal birth. This method is believed to help establish a healthy gut microbiome in the newborn, which may contribute to the development of a robust immune system and has been shown to fully restore the skin and oral microbial compositions to the vaginally delivered profiles within 72 hours (Mueller et al., 2023). Maternal FMT administered orally achieved 89% similarity in the infant gut microbiota to that of the vaginal birth cohort by 3 months postintervention, with no serious adverse events reported (Korpela et al., 2020). Maternal vaginal secretions may carry pathogens and cause neonatal infections (Lee et al., 2017). Additionally, their long-term effect on gut microbiota remodeling is limited and the evidence is inconsistent (Liu et al., 2023). Therefore, current mainstream medical organizations recommend that vaginal seeding be performed only within research protocols approved by the Institutional Review Board (IRB), and should not be carried out in routine clinical or home settings (Wharton and Birsner, 2017). Maternal fecal microbiota transplantation (FMT) application in cesarean section neonates is even more preliminary. A small number of case reports have shown that oral administration of maternal fecal microbiota capsules can partially reconstitute the gut microbiota of cesarean section infants (Liu et al., 2024), but long-term follow-up data are completely lacking. Neonatal FMT faces risks including occult pathogen transmission, transfer of antibiotic resistance genes, abnormal activation of the immature immune system, and unknown risks of long-term autoimmune/metabolic diseases (Korpela et al., 2020b; Limaye and Ratner, 2020; Sassin et al., 2022). It is only permitted to conduct exploratory studies under strict ethical supervision.

### 5.3 Application of probiotics and prebiotics

The therapeutic potential of probiotics in neonatal care after birth is strongly supported by clinical evidence. A network meta-analysis encompassing 106 trials (n = 25,840 preterm infants) identified multistrain formulations containing *B. infantis* as the most effective probiotic, reducing all-cause mortality by 31% (RR 0.69, 95% CI 0.54–0.88) and necrotizing enterocolitis incidence by 45% (RR 0.55, 95% CI 0.41–0.73) (Wang et al., 2023b). Furthermore, microbiota-directed precision nutrition shows promise for addressing malnutrition. In a phase 2 RCT (n = 123), children aged 12–18 months receiving MDCF-2 presented *Prevotella*-enriched gut microbiota (LEfSe LDA score = 4.1) alongside improved growth and health outcomes (Chen et al., 2021). The impact of early-life microbial modulation extends beyond the gut, leveraging the gut-lung axis to influence respiratory health. Clinical studies demonstrate that early postnatal supplementation with prebiotics or probiotics significantly reduces the risk of viral respiratory infections and exerts preventive effects against asthma and allergic disorders in infants (Luoto et al., 2014; Uwaezuoke et al., 2022). This provides a compelling paradigm for using gut-centric strategies to prevent common childhood respiratory conditions. However, the effect size of these studies is small, and there is a risk of publication bias (Agha et al., 2023). High-dose probiotics have occasionally been reported to cause probiotic-related sepsis in very low birth weight infants (Alshaikh et al., 2025); therefore, a strict risk-benefit assessment must be conducted before use.

Despite remarkable advances in microbiome research, the translation of microbiome-based therapies faces regulatory challenges that lag behind scientific discoveries. Strain-level functional heterogeneity, geographic variations in microbial composition, and host-specific interactions complicate the standardization of microbial biomarkers for diagnostic or therapeutic use. Future research must unravel host–microbe interaction mechanisms and pivot towards precision medicine. A key frontier is the development of microbial biomarkers for risk stratification, enabling targeted interventions for infants most likely to develop specific immune or metabolic disorders. Overall, the clinical translation of microbiome research in early life holds immense promise but is still in its nascent stages; clinical translation is still accompanied by significant potential risks and ethical considerations, which must be fully emphasized and addressed in future research and clinical practice.

In summary, the clinical translation of early-life microbiome research holds great potential, but it is still in its infancy. The vast majority of intervention measures should be regarded as research tools rather than mature clinical treatment methods. Future research should prioritize the implementation of well-designed randomized controlled trials (including long-term safety follow-up), establish standardized operating procedures, develop microbial biomarkers for predicting therapeutic efficacy, and promote the improvement of regulatory frameworks. Only after fully addressing the aforementioned challenges can microbiome-targeted therapies be safely and effectively applied in perinatal clinical practice.

## 6 Conclusion

Alongside the establishment and development of organs, the microbiota undergoes significant changes within the first 1,000 days of life. The microbiota is subject to influence and regulation by the local and systemic environment of an infant. Alterations in microbial communities, which may arise from environmental factors or disease states, can significantly impact the health and clinical outcomes of certain diseases. However, further understanding of these outcomes is limited by current study design, detection methods (e.g., culture-independent sequencing, multiomics), and sampling challenges. Therefore, comprehensive research employing advanced experimental and sampling techniques is essential to uncover the intricate compositions of the microbiota and to understand the intricate mechanisms and networks by which they exert their influence on health and disease. Research into the human microbiome holds the promise of enhancing long-term health outcomes and fostering the creation of innovative treatments for various diseases.
